# Serum Selenium Status and Its Interrelationship with Serum Biomarkers of Thyroid Function and Antioxidant Defense in Hashimoto’s Thyroiditis

**DOI:** 10.3390/antiox9111070

**Published:** 2020-10-31

**Authors:** Rahim Rostami, Sarmad Nourooz-Zadeh, Afshin Mohammadi, Hamid Reza Khalkhali, Gordon Ferns, Jaffar Nourooz-Zadeh

**Affiliations:** 1 Department of Clinical Biochemistry and Nutrition, Urmia University of Medical Sciences, 5714783734 Urmia, Iran; rostami.r@umsu.ac.ir; 2 Faculty of Medicine, University of Medical Sciences, 5714783734 Urmia, Iran; nouroozzadeh.s@umsu.ac.ir; 3 Department of Radiology, Urmia University of Medical Sciences, 5714783734 Urmia, Iran; mohammadi.a@umsu.ac.ir; 4 Department of Biostatistics and Epidemiology, Urmia University of Medical Sciences, 5714783734 Urmia, Iran; khalkhali@umsu.ac.ir; 5 Department of Medical Education Brighton and Sussex Medical School, England BN1 9PX, UK; G.Ferns@bsms.ac.uk; 6 Nephrology and Kidney Transplant Research Center, Urmia University of Medical Sciences, 5714783734 Urmia, Iran

**Keywords:** selenium, Hashimoto’s thyroiditis, glutathione, glutathione peroxidase, iodine, thyroid hormones, oxidative stress

## Abstract

Selenium (Se) deficiency has been implicated in the pathogenesis of Hashimoto’s thyroiditis (HT), although the available evidence is limited. The present study aimed to explore the interrelationships between serum Se status with measures of thyroid function and antioxidant defense in new cases of HT patients with hypoechogenic thyroid. HT patients (*n* = 49) and matched controls (*n* = 50) were recruited. Selenium, thyroid hormone panel, thyroid volume (TVol), glutathione (GSH), glutathione peroxidase3 (GPx3) activity, urinary iodine concentration (UIC), and urinary creatinine (Cr) were assessed. HT patients exhibited lower Se levels compared to controls (*p* < 0.001) with the rates of Se-deficient (<0.85 µmol/L) participants being 58.8% and 34%, respectively. Se-deficient patients exhibited higher thyroid stimulating hormone (TSH), Thyroid volume (TVol), thyroglobulin, antibody-titers, GPx3 activity and UIC/Cr compared to Se-sufficient patients (all *p* < 0.001). In the Se-deficient patients, inverse correlations were seen between Se-levels with TSH, TVol, and Thyroid peroxidase antibody (TPO-Ab) (all *p* < 0.001). This study is the first to uncover that coexisting Se-deficiency and elevated iodine in HT may enhance autoimmune reactions and accelerate the deterioration of thyroid function through oxidative stress. Our study also highlights the importance of optimal Se status in this disease, thus providing a rationale for the execution of intervention trials for the evaluation of the clinical benefits of antioxidant-status improvement in HT.

## 1. Introduction

Bodily functions are controlled through an intricate interplay between the nervous and hormonal systems. A key player in the regulation of metabolism is the thyroid gland. Located in the anterior neck, the gland may exhibit both hypo- and hyperactivity arising from myriad etiologies ranging from viral infections to dietary insufficiencies. Hashimoto’s thyroiditis (HT) is the most prevalent cause of chronic hypothyroidism in iodine sufficient regions, which stems from an autoimmune response against thyroid peroxidase (TPO) and/or thyroglobulin (Tg) [1,2,3]. With a global prevalence of 5% among all ages and sexes, HT is 4–10 times more prevalent in women than in men. The reported regional prevalence of HT is variable, mainly due to the application of different diagnostic criteria, laboratory testing methods and global localization [4]. The complex etiology of HT involves the partially understood interplay between the immune system, oxidative stress and trace element imbalance [1,5,6,7].

Selenium (Se) is an essential trace element, vital for the proper function of both the thyroid and the immune system [8,9]. Selenium exercises its impact on thyroid regulation mainly in the form of selenoproteins, a large family of enzymes highly influential on the oxidative balance, cell proliferation and differentiation and the production of thyroid hormones [10]. Dietary intake is the main contributor to selenium store replenishment and the selenium content of various foods varies in accordance with soil selenium abundance. Foods rich in selenium content include Brazil nuts, eggs, meats and various grains [11,12,13,14,15]. Selenium levels in the drinking water of the majority of the population around the globe is not considered nutritionally significant. In addition, it has been reported that smoking has a negative impact on body selenium stores. Inadequate selenium intake results in selenium deficiency which is estimated to affect between 500 million and a billion individuals, placing this population at an increased risk for an array of autoimmune disorders. Individuals with depleted selenium stores are incapable of washing out the oxidative stress imposed by the production of thyroid hormones which, in association with other etiologies, results in higher chances of developing HT. Contempre et al. [16] previously reported that Se supplementation was associated with significant improvement in thyroid hormone profile in clinically euthyroid- or hypothyroid subjects, hence suggesting the potential role of Se-deficiency in the pathophysiology of thyroid dysfunction. This treatment resulted in a marked reduction in serum glutathione peroxidase (GPx3) activity and an improvement in thyroid function. Observational studies have also revealed associations between impaired Se-status and an increased risk of thyroid dysfunction [17]. The exact mechanisms behind the adverse influence of Se-deficiency in HT remain to be elucidated. However, it has been proposed that diminished Se stores may negatively impact the endocrine system by inducing an oxidant–antioxidant imbalance, as well as activating the immune system against the host [18]. In addition to hormonal dysfunction, such imbalance may also cause further complications. For instance, Mertere et al. reported an association between decreased GPx1 activity and thyroid cancer [19].

Information regarding the relationship between serum Se-levels and measures of antioxidant defense is limited and inconclusive. Przybylik-Mazurek et al. [20] failed to uncover differences between HT patients and controls when assessing the Ferric Reducing Ability of Plasma (FRAP), serum Se-levels and GPx3 activity. Nourbakhash et al. [21] sought to identify a relationship between serum levels of Se, selenoprotein p (SePP) and GPx3 activity in children and adolescents with HT receiving levothyroxine; they reported a lack of significance regarding the differences present between the two groups.

The current study aimed to investigate the interrelationships between serum Se levels and indices of thyroid function, antibody titers, GPx3 activity, reduced glutathione (GSH) and urinary iodine excretion (UIC) in new cases with HT who were found to have a hypoechogenic thyroid on ultrasound evaluation.

## 2. Materials and Methods

### 2.1. Studied Population

**Figure 1:**
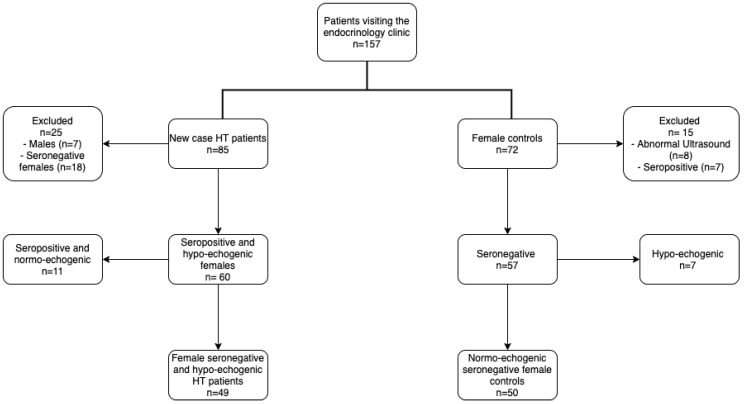
Patient and control enrollment process flowchart.

For the purpose of this study, a total of 157 female patients visiting the endocrinology clinic of Imam Khomeini Teaching Hospital (Urmia, Iran) for the first time, regardless of reason, were evaluated for enrollment through convenience sampling. Forty-nine new cases of HT (range: 13–47 years) and 50 controls (range: 12–52 years) were sequentially recruited from this population. Exclusion criteria for the HT group were as follows: (1) treatment with anti-thyroid therapy within 6 weeks prior to study initiation; (2) multivitamin and/or trace element supplementation; (3) menopause or irregular menstrual cycle; (4) a history of smoking; (5) a recent history of dieting; (6) pregnancy or lactation; (7) diabetes mellitus. Healthy volunteers with no history of thyroid dysfunction, with normal thyroid ultrasound echogenicity and with negative serologic antithyroid antibody evaluations were enrolled as controls. The following exclusion criteria were considered for the control group: (1) a history of chronic illness; (2) evidence of inflammation or autoimmunity; (3) a history of thyroid dysfunction; (4) multivitamin and/or trace element supplementation; (5) irregular menstrual cycle; (6) smoking; (7) dieting or fasting; (8) pregnancy or lactation; (9) diabetes. A flow chart of the recruitment process for both patients and controls is presented in Figure 1. Selenium deficiency was defined as a serum <0.85 µmol/L [22]. The study was approved by the Ethics Committee of Urmia University of Medical Sciences, Urmia, Iran (Ethics code: IR.umsu.rec.1388.32). Informed written consent was obtained from all participants prior to study entry.

### 2.2. Blood Collection

Blood samples (5 mL) were collected by venipuncture, allowed to stand for 10 min at room temperature and subsequently centrifuged at 1000 rpm for 15 min. Serum aliquots (250 µL) were transferred to Eppendorf tubes and kept at −70 °C until analysis.

### 2.3. Urine Collection

Fasting urine samples (10 mL) were collected. Aliquots (1 mL) were transferred to Eppendorf tubes and stored at −70 °C until analysis.

### 2.4. Assessment of Thyroid Volume and Thyroid Echogenicity

Thyroid volume (TVol) was determined using a 7.5 MHz linear transducer real-time ultrasonography (Nemio30, Toshiba, Tokyo, Japan). A senior radiologist performed the assessments while patients were in a supine position with their neck in hyperextension. Total thyroid volume, which refers to the sum of both lobes, was calculated using the following formula: width × length × depth × 0.479 for each lobe [23]. Echogenicity was determined by comparing the echo-intensity of the thyroid parenchyma to the surrounding neck musculature. The isthmus was excluded to avoid the interference of reflecting waves from the tracheal cartilage [24].

### 2.5. Thyroid Function and Antibody Tests

Thyroid stimulating hormone (TSH), T_4_, T_3_, T_3_-uptake, fT_4_, and fT_3_ were assessed by ELISA (Pishtaz Teb, Tehran, Iran). TPO-Ab and Tg-Ab titers, as well as thyroglobulin levels were assessed by enzyme immunoassay (EIA; AESKU Inc, Hamburg, Germany). According to the manufacturer’s information, Thyroid peroxidase- antibody (TPO-Ab) levels ≤40 IU/m, between 40–60 IU/mL and >60 IU/mL were defined as negative, equivocal and positive, respectively. Suggested values for negative, equivocal and positive thyroglobulin-antibody (Tg-Ab) were ≤120 IU/mL, 120–180 IU/mL and >180 IU/mL, respectively. According to the manufacturer’s manual, a normal range for thyroglobulin was defined as 50 μg/L.

### 2.6. Selenium Assay

Serum Se was determined by atomic absorption spectrophotometer (GF-AAS; PG 990, England) equipped with a Se hollow cathode lamp and pyrolytically coated graphite tubes with a stabilized temperature platform furnace. Argon was used as the required pure gas. All glassware were acid-washed (10% nitric acid) and rinsed thoroughly with deionized water before use. Modifiers, reagents, calibrators and standards were prepared according to the National Health and Nutrition Examination Survey protocol [25]. The signal was read at 196.0 nm at a slit width of 0.7 nm [26]. Trace Elements Serum (Seronorm™, Sero, Billingstad, Norway) was used as a calibrator.

### 2.7. Assessment of Urinary Iodine Excretion (UIC)

Measurement of UIC was carried out using the Sandell-Kolthoff reaction, as described in detail elsewhere [27,28]. In brief, urine samples were thawed at room temperature, vortexed to release particulates and subsequently centrifuged at 15,000 rpm for 5 min. One milliliter aliquots of ammonium persulfate solution (1 mol/L in 2 N sulfuric acid) were transferred into 10 mL glass-tubes to which 250 µL aliquots of the urine samples were added. The samples were rapidly vortexed, heated at 100 °C for 60 min and allowed to stand at room temperature for 10 min. A 300 μL aliquot of cerium (IV) ammonium sulphate (VI) solution (0.034 mol/L in 2N sulfuric acid) was added to the solution. Immediately after mixing, light absorbance at 405 nm was measured at 10 s intervals over a duration of 2 min using a double beam UV/Vis Perkin Elmer spectrophotometer.

### 2.8. Measurement of Urinary Creatinine (UCr)

Measurements for urinary creatinine were performed using the Jaffe method (Pishtaz Teb, Tehran, Iran) on 1/50 diluted urine samples using a BT-1500 auto-analyzer.

### 2.9. Measurement of Serum Glutathione Peroxidase (GPx3) Activity

The reaction mixture (950 μL) for the determination of serum GPx3 activity comprised Tris buffer (50 mmol/L; PH 7.6), Na_2_EDTA (1 mmol), GPx (2 mmol), NADPH (0.2 mmol), sodium azide (4 mmol) and glutathione reductase (1000 U). The reaction blend was mixed with the serum sample (50 μL). This solution was incubated at 37 °C for 5 min and subsequently mixed with H_2_O_2_ (8.8 mmol/L; 10 μL). Absorbance was read at 340 nm, continuously, over 3 min using a double beam UV/Vis Perkin Elmer spectrophotometer [29].

### 2.10. Glutathione Determination

For glutathione determination, serum (100 μL) was mixed with sulfosalicylic acid 5% (100 μL) and subsequently centrifuged for 10 min at 1000 rpm. The supernatant (50 μL) was mixed with phosphate-EDTA buffer (0.1 mol/0.001 mol; pH: 7.4; 200 μL), NADPH (2 mg/mL in 0.1 mmol/L KOH; 100 μL), DTNB (1.5 mg/mL in 0.5% NaHCO_3_; 20 μL) and glutathione reductase (6 U/mL in 0.1 mol/0.001 mol phosphate-EDTA buffer; 20 μL). Absorbance was read at 412 nm using a double beam UV/Vis Perkin Elmer spectrophotometer [29]. All chemicals and reagents were obtained from Merck (Darmstadt, Germany)

### 2.11. Statistical Analysis

Statistical analysis was carried out on SPSS software package for Windows, v. 16.0 (IBM, Chicago, IL, USA). Data were either expressed as mean ± SD or median. Differences between groups were evaluated with nonparametric tests and considered significant if *p* < 0.05. Pearson’s correlation coefficient was used to evaluate associations between variables. Nonparametric variables are presented as median (min–max range). Numeric variables were analyzed using Student’s *t*-test or the Mann-Whitney U-test (for skewed data), while categorical data were analyzed using the *χ*^2^ test. One way analysis of variance (ANOVA) was used to compare parameters between selenium sufficient and deficient HT patients and controls. Skewed variables were natural logarithm-transformed. To test the hypothesis that serum Se levels are significantly different between HT subjects with selenium- deficiency or sufficiency (power: 80%; confidence interval: 95%), sample size was calculated as described elsewhere [30]. Minimum sample requirement per group was estimated to be 50. Whisker plots were created using Prism version 8.0.0 for Windows (Graphpad, La Jolla, CA, USA).

## 3. Results

### 3.1. Population Characteristics and Hypothyroidism

After considering eligibility and exclusion criteria, a total of 49 new case HT patients and 50 healthy controls were enrolled (Table 1). As defined by FT_4_ and TSH levels of <9.01 pmol/L and >5.2 mIU/L respectively, none of the controls were hypothyroid. However, 36 (73.5%) and 13 (26.5%) of the HT patients had subclinical and clinical hypothyroidism, respectively.

### 3.2. Comparison of Biochemical Paraameters in Hashimato Thyroiditis Patients and Controls

**Figure 2:**
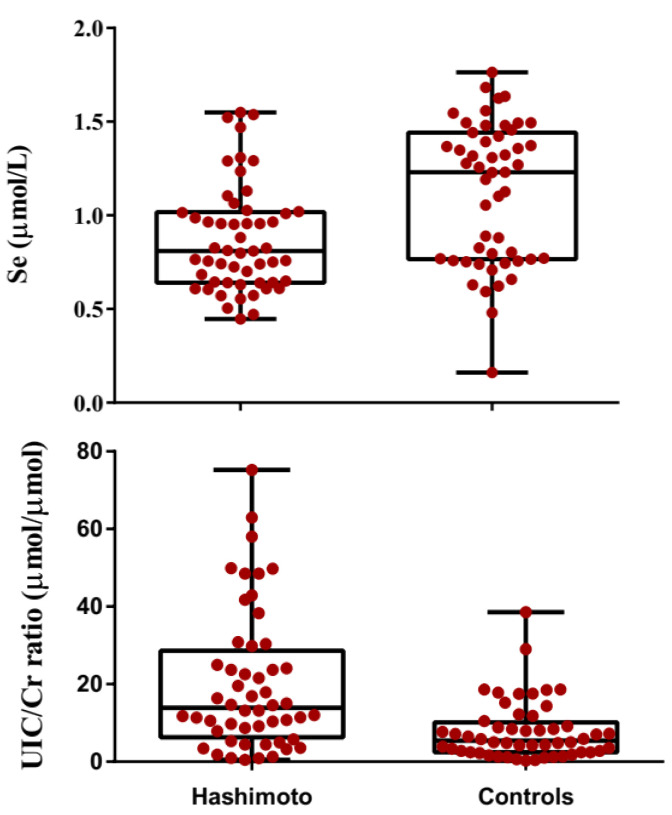
Box and whisker plots showing the selenium contents and UIC/Cr in HT patients and controls. The box describes first, second (median) and third quartiles. The whisker length represents minimum to maximum range. Each dot represents a single participant.

In the patient group as a whole, mean serum Se levels was lower than that of the controls (0.87 ± 0.29 μmol/L vs. 1.11 ± 0.37 μmol/L; *p* < 0.001). Box and whisker plots showing serum Se-levels in Hashimoto patients and matched controls are displayed in Figure 2. Mean TVol was greater in the patients with HT compared to the controls (median: 14.4 mL; range: 4.20–40 mL vs. median: 9.35 mL; range: 2.1–19 mL; *p* < 0.001). An individual comparison of lobes for the controls revealed that the mean left- and right- lobe volumes were 5.66 ± 2.98 and 4.76 ± 2.43 mL (5.0 mL; 1.2–21 mL vs. 4.2 mL; 0.9–16.0 mL), respectively. The respective values for the HT patients were 8.10 ± 4.43 mL (7.5 mL; 1.5–26 mL) and 7.23 ±4.41 mL (6.5 mL; 1.5–28 mL). The patients group also had lower GSH, T_4_, T_3_, FT_3_ and FT_4_ when compared to the controls (all *p* < 0.001). On the other hand, thyroglobulin, GPx3 activity, TSH, T_3_-uptake, TPO-Ab, Tg-Ab and UIC/Cr were higher in the patient group than the controls (all *p* < 0.001). Clinical and biochemical characteristics of controls and HT patients are presented in Table 1. Box and whisker plots showing serum Se-levels and UIC/Cr in Hashimoto patients and matched controls are displayed in Figure 2.

**Figure 3:**
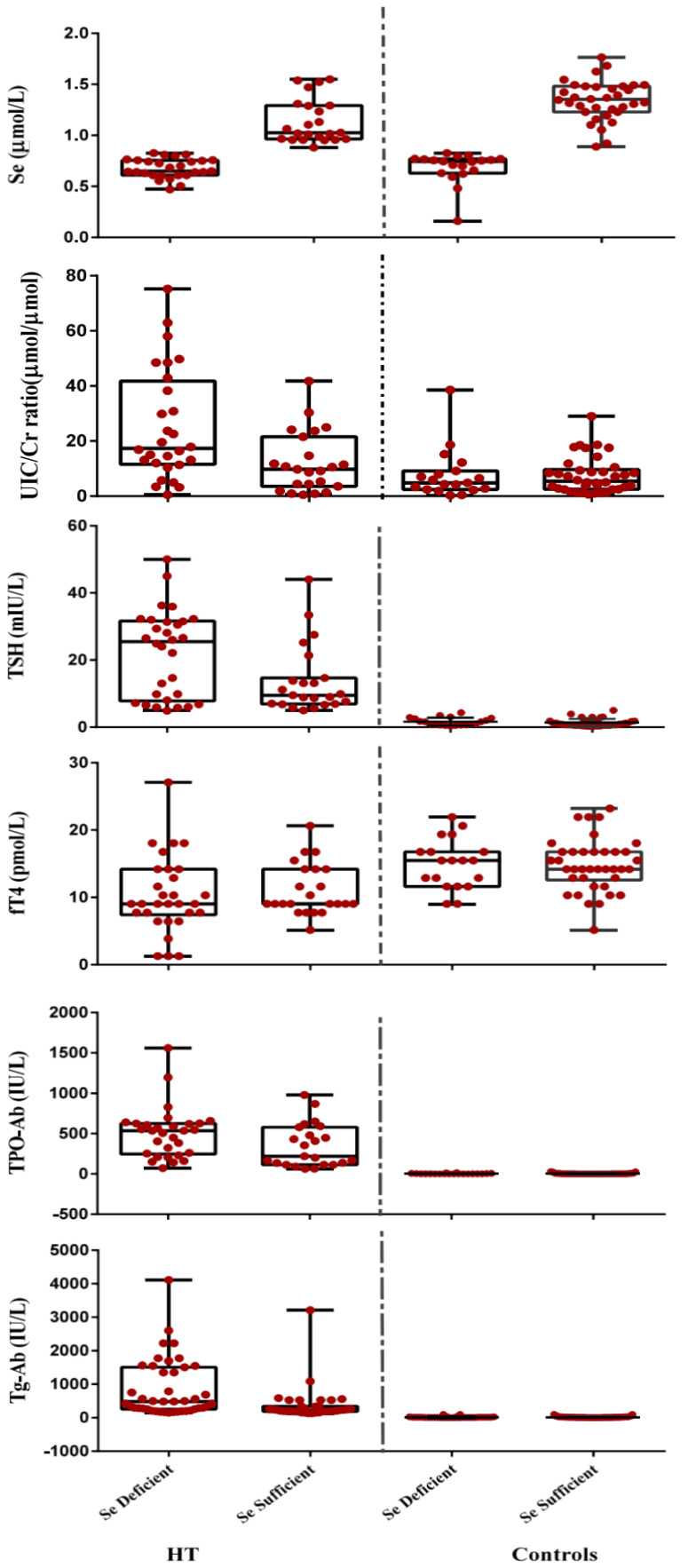
Box and whisker plots showing the levels of selenium, UiC/Cr, TSH, FT_4_, TPO-Ab and Tg-Ab HT in patients and controls (sufficient: >0.85 µmol/L and deficient: <0.85 µmol/L). The box describes first, second (median) and third quartiles. The whisker length represents minimum to maximum range. Each dot represents a single participant.

Analysis using one-way ANOVA revealed a significant difference between the Se- deficient and Se-sufficient HT patients with the controls for the majority of the parameters, except BMI (*p* = 0.155). Further comparison using the Tukey’s HSD post hoc analysis revealed that the differences between the mean TSH, T4, T3, FT4, FT3, T3 uptake, Tg, TVol, TPO-Ab, Tg-Ab, GSH, GPx3 and UIC values were not significant between the Se-sufficient and Se-deficient controls. However, a significant difference was observed regarding TSH, TPO-Ab, Tg-Ab, and TVol values between selenium sufficient and deficient HT patients (*p* = 0.019, 0.02, 0.35, and 0.042). Figure 3 shows box and whisker plots for selenium, UIC/Cr TSH, FT4 and TPO-Ab in Hashimoto patients and matched controls divided according to serum selenium status (sufficient: >0.85 µmol/L and deficient: <0.85 µmol/L).

### 3.3. Correlations

We found that negative associations were present between serum Se and TVol, TSH, TPO-Ab or UIC (r = −0.427; r = −0.422; r = −0.387; r = −0.360; all *p* < 0.05). Significant positive correlations were also observed between TSH and TPO-Ab; TVol and TPO-Ab; TVol and TSH (r = 0.574; r = 0.514; r = 0.803; all *p* < 0.05). Inverse associations were seen between GSH and TPO-Ab. TSH, TVol or thyroglobulin (r = −0.263; r = −0.367; r = −0.387; r = −0.283, r = −0.288; all *p* < 0.05).

In the control group, mean Se levels in the selenium- deficient and sufficient participants were 0.69 ± 0.16 µmol/L and 1.33 ± 0.22 µmol/L (*p* < 0.001) (0.74; 0.16–0.83 vs. 1.35; 0.88–1.76 µmol/L). The Se-deficient controls had a slightly larger TVol and higher serum thyroglobulin level compared to Se-sufficient participants.

In the HT group, mean serum Se levels in the deficient- and sufficient participants were 0.67 ± 0.11 µmol/L and 1.16 ± 0.22 µg/L (*p* < 0.001) (0.65; 0.45–0.83 vs. 1.03; 0.88–1.55). The Se-deficient patients exhibited significantly higher thyroglobulin, TVol, TSH, TPO-Ab and Tg-Ab activity and UIC/Cr were than those for Se-sufficient patients. No differences were seen with respect to GSH content and GPx3 activity.

Negative associations were observed between serum Se with TVol, TPO-Ab and TSH in Se deficient HT patients (r = −0.324; r = −0.412; r = −0.331; all *p* < 0.05). Positive correlations were observed between serum TSH and TPO-Ab; TVol and TPO-Ab; TVol and TSH (r = 0.550: r = 0.539; r = 0.790; all *p* < 0.05).

## 4. Discussion

It has been proposed that Se-deficiency in HT patients is associated with an array of adverse effects, such as compromised antioxidant state, increased expression of harmful epitopes and apoptosis [8,9,31]. However, the absence of adequate laboratory evidence has hampered the clarification of the underlying molecular mechanisms. This investigation attempted to explore the interrelationships between Se-status with indices of thyroid function and antioxidant state in new case HT patients with a hypoechogenic thyroid.

In this investigation, the mean Se level for the controls was 1.11 ± 0.37 μmol/L, which is similar to those reported in other countries including Australia, Austria, Brazil, England, and Turkey [32,33,34,35,36]. In the HT patients as a whole, the mean Se-level was 22% lower compared to that of the control group. The extent of difference in Se levels between HT patients and the controls is in agreement with previous investigations reporting diminished Se-status in other inflammatory diseases [18,20,37,38,39]. Wimmer et al. [37] compared the Se-level of autoimmune thyroiditis patients with matched controls. In their study, 12% of the HT patients were selenium deficient (≤1.02 μmol/L). The remaining (88%) had adequate selenium levels. Data regarding the respective frequencies in the control group were not published. In the present investigation, segregation of the control group according to Se-status (<0.85 μmol/L versus >0.85 μmol/L) uncovered that mean Se level in deficient individuals was 52% lower than the sufficient subgroup. Interestingly, 34% of the controls were selenium deficient. The mean selenium level in the selenium deficient controls was in good agreement with those reported for a Chinese population residing in an iodine-sufficient area [17,40]. In the HT group, mean Se levels in the deficient subgroup was 43% lower than that for the respective Se-sufficient subset. Of note is that the frequency of Se-deficient HT patients is markedly higher than that of Se-deficient controls (58.8%). The divergence regarding the prevalence of individuals with- and without Se-deficiency between the studied populations is not explainable by dietary habits since the participants were matched with respect to ethnicity and residence. A possible explanation for the difference in the frequency of Se-deficient individuals between HT patients and controls is that the variation may reflect differences in the efficiency of selenocysteine incorporation into proteins in health and disease. This finding will have an important implication for the study of Se-deficiency and its correction in the prevention thyroid dysfunction in subjects with- and without pre-existing autoimmune dysfunction.

In the controls, Se-deficiency had no impact on indices of antioxidant defense, as evidenced by serum GSH and GPx3 activity, antithyroid antibody titers, thyroid hormone panel or UIC. However, Se-deficiency coincided with marginal increases in TVol and serum thyroglobulin. A possible explanation may be that coincident Se-deficiency and borderline iodine insufficiency stimulate an increased colloid accumulation and glandular secretion of thyroglobulin by a mechanism independent of TSH regulation [41]. Derumeaux et al. [42] found an inverse association between serum Se concentration and TVol in mildly iodine deficient French females (*n* = 1108; 35–60 years old) with suboptimal Se-status (i.e., 1.10 µmol/L). On the other hand, Ozata et al. [43], found no relationship between serum Se levels with TVol in a Turkish population residing in a region with moderate iodine deficiency. Possible explanation(s) for the divergence between our study and the previous investigations may be a consequence of the differences in Se-iodine balance and/or population inclusion criteria. Further studies with larger sample sizes are necessary to ascertain the interrelationship between Se-deficiency and TVol in healthy individuals.

In the HT patients, Se-deficiency coincided with higher TSH, TPO-Ab, Tg-Ab and larger TVol when compared to the Se-sufficient patients. Wimmer et al. examined the association between serum Se levels with antibody titers and thyroid hormone panel in a population of HT patients [37]. No differences were seen in Se-levels when patients were segregated according to TPO-Ab (>500 U/mL versus <500 U/mL). A study involving HT patients (*n* = 80) not receiving thyroid medication at the time of study initiation, reported a marked reduction in TPO-Ab titer following a 12-month supplementation with sodium selenite (80 μg/day) [44]. The decrease in TPO-Ab titer was found to be more pronounced in patients with higher baseline antibody titers. This investigation provides a rationale for the execution of new intervention trials for the evaluation of the clinical benefits of Se-status improvement in HT [18]. In contrast and in a study on 18 HT patients supplemented with 200 micrograms sodium selenite over a period of 3 months, Karanikas et al. failed to observe a significant difference in TPO-Ab levels compared to baseline and placebo (524 ± 452 vs. 505 ± 464 IU/mL; *p* > 0.05) [45].

This investigation reports that GSH levels in Se-sufficient HT patients was 64% lower than that of the respective controls. Of note is that no further fall in GSH content was observed due to the manifestation of Se-deficiency in the HT patients. GSH is a major intracellular hydro-soluble antioxidant agent [46,47]. Cells of the immune system contain polyunsaturated fatty acids which are highly susceptible to the damaging effects of respiratory burst [48]. The lipid peroxides resulting from polyunsaturated fatty acid oxidation act as a generator of reactive oxygen species (ROS) which eventually lead to GSH depletion. ROS also activates multiple stress kinase pathways and redox sensitive transcription factors, including nuclear factor kappa-light-chain-enhancer of activated B cells (NF-κB) and Activator protein 1 (AP-1) [49,50,51]. GSH also is necessary for antigen unfolding by forming disulphide bonds with the antigen [52]. GSH plays a key role in the maintenance and regulation of certain immunological functions including the activation of lymphocytes and functional activity of NK cells [53,54]. The resulting lipid peroxides act as a generator of (ROS) and depletion of GSH. Taken together these lines of evidence imply that the native immune response may fail if tissue GSH levels remain low for a long time. A recent study by Richie et al. has shown that daily consumption of GSH supplements (250 or 1000 mg/day) for 6 months was effective at increasing body compartment stores of GSH [55]. The treatments were also associated with enhanced NK cell cytotoxicity. It remains to be investigated if this rationale is an approachable strategy to reduce oxidative stress-induced inflammation in HT patients.

The physiological functions of GPx3, a seleno-cysteine protein, comprise the regulation of thyroid hormone synthesis by H_2_O_2_ tuning and protection against oxidative damage. Several research groups have found both high and low levels GPx3 activities in HT when compared to control subjects. A possible reason for the divergence between the reports may be due to the variability and complexity of the regulating mechanism of oxidative stress in humans. In the present investigation, the GPx3 activity of the entire patient group was 22% higher compared to the control group. Interestingly, the increase in GPx3 activity was equally pronounced in the patient with- and without Se-deficiency. The lack of a substantial alteration in GPx3 activity in the Se-deficient patients implies that GPx3 is probably performing beyond its Vmax (i.e., lower capacity to neutralize accumulated H_2_O_2_) [29]. The evidence for this postulation is the lower GPx3 activity in the patients with optimal Se-intake when compared to those with Se-deficient patients. Increased H_2_O_2_ production will subsequently deplete GSH as the first line of defense against oxidative stress leading to impaired GSH recycling. In a balanced redox state, oxidized glutathione (GSSG) will be exported from the mitochondria to the cytosol where it is restored to its reduced form (GSH) by glutathione reductase. Then, GSH can either return to the cell or be utilized as a cofactor for GPx [56]. Impaired GSH recycling will result in oxidative damage to membrane lipids, proteins and DNA that leads to cell death by necrosis or apoptosis. Taken together, the results imply that the modulation of defense mechanisms in patients with Se-deficiency results in the abnormal iodination of certain proteins, leading to cell apoptosis and/or the increased risk of exposure to unusual epitopes that are easily recognized, and reacted to, by the immune system.

Another important finding from the present investigation is that the UIC/Cr ratio of the Se-deficient patients was 59% higher compared to the respective Se-sufficient HT patients. The cause(s) for the higher iodine retention in Se-deficient HT patients is unexplained. However, it is likely to be a compensation response to thyroid hormone biosynthesis. Excess iodine in HT patients mediates tissue injury by an array of mechanisms, the most important of which are (1) chemokine-mediated lymphocytic infiltration in the thyroid parenchyma; (2) increased thyroglobulin iodination and induction of NF-κB -dependent expression of proinflammatory markers such as ICAM-1 and IL-6; (3) increased polyunsaturated fatty acid auto-oxidation followed by the generation of an array of aldehydes such as 4-hydroxy-2-alkenals (4-HNE). Interaction between 4-HNE and native proteins yields aldehyde-protein adducts with the capability to induce pathogenic antibodies) [4,57,58]. These events form an augmentation loop that culminate in increased antigen presentation against TPO and thyroglobulin, leading to increased oxidative stress. Another explanation for the optimum iodine status may be that Se deficiency might have, by a yet to be identified mechanism, improved iodine uptake despite the limited supply. However, this hypothesis requires extensive and fundamental research. It has been well documented that excessive iodine intake, especially after a period of iodine deficiency, is associated with an increased risk for thyroid autoimmunity. Proposed mechanisms for this include the creation of thyroglobulin with neoantigenic determinants unrecognized by the immune system, increased thyroid antigen release from cells undergoing apoptotic and necrotic changes [59] and thyroid cell membrane damage due to sabotaged oxidative stress as well as increased iodine and oxygen radicals [60]. The inverse associations between Se-levels and TPO-Ab, TVol, and TSH in Se-deficient patients provides the first clinical basis for the hypothesis that altered iodine-Se status could be responsible for the induction of complement-mediated damage in HT [61]. The importance of iodine-Se balance in therapeutic intervention with Se demands further research.

A number of studies have set out to evaluate the impact of selenium supplementation in Hashimoto’s thyroiditis. In a study on 192 HT patients, Pirola et al. [62] supplemented 96 patients with 83 micrograms of selemomethionine/day over a period of 4 months, without additional dietary modifications. Selenium supplementation restored euthyroidism in one third of the patients, while only three participants of the control group became euthyroid over the course of the study (ratio of 10:1). Of note, TPO-Ab was significantly reduced in the supplemented patients, regardless of response. They also reported improved thyroid echogenicity following selenium supplementation. Wang et al. [63] executed a double-blind, randomized controlled trial in which a total of 364 patients were divided based on overt hypothyroidism and a total of 181 patients were randomly assigned to receive high selenium yeast while the remainder received placebo yeast. They reported a significant decline (10.7% and 10.0%) in TPO-Ab after 3 and 6 months of supplementation, while this marker showed gradual elevation in the placebo groups (*p* < 0.001). Esposito et al. [64] enrolled a cohort of 76 euthyroid HT patients and supplemented the population either with selenomethionine or placebo for a period of 6 months. They reported that although FT_4_ and FT_3_ levels decreased and increased, respectively, in the selenomethionine group over the study period (*p* < 0.04 and <0.03). No significant differences were present regarding TSH, FT_4_, FT_3_, TPO-Ab values and thyroid echogenicity in this group compared to the controls at any of the dedicated time points.

## 5. Conclusions

In conclusion, we found that the frequency of Se-deficiency in HT patients and healthy controls was 58.8% and 34%. In the controls, Se-deficiency failed to exert an impact on antioxidant defense, as evidenced by GSH and GPx3 activity, although it was associated with increased TVol and thyroglobulin levels. In the patients with HT, Se-deficiency was coincident with increased whole-body iodine store, GPx3 activity, TVol, TPO-Ab, Tg-Ab and TSH compared to Se sufficient participants. These findings suggest that in the setting of optimal iodine status, Se-deficiency plays an important role in the immunological events leading to the induction of the deleterious cycle of oxidative stress and subsequent thyroid failure.

Our findings highlight the importance of optimal Se status in Hashimoto’s thyroiditis, thus providing a rationale for the execution of intervention trials for the evaluation of the clinical benefits of improvement of antioxidant status in HT. Further prospective studies with and without controlled dietary modifications are required to further cement the findings of our study. Such knowledge will better arm clinicians for the optimal management of HT by taking the various factors in play into consideration.

## Figures and Tables

**Table 1:** Clinical- and biochemical characteristics in new case Hashimoto patients with hypoechogenic thyroid and matched controls.

Variables.	Hashimoto’s Thyroiditis (*n* = 49)	Control (*n* = 50)	*p*-Value
Age (years)	33.77 ± 10.46	33.32 ± 9.98	NS
BMI (Kg/m^2^)	28.53 ± 8.27	26.34 ± 5.42	NS
TSH (mIU/L)	18.57 ± 12.46	1.41 ± 1.17	<0.001
T_4_ (nmol/L)	84.4 ± 33.3	127.8 ± 20.4	<0.001
T_3_ (nmol/L)	1.9 ± 0.84	2.9 ± 0.78	<0.001
fT_4_ (pmol/L)	10.4 ± 4.89	14.8 ± 3.86	<0.001
fT_3_ (pmol/L)	4.5 ± 1.18	5.6 ± 1.01	<0.001
T3up (%)	30.51 ± 2.07	28.71 ± 3.58	<0.001
Tg (μg/L)	23.63 ± 19.63	11.85 ± 12.41	<0.001
TVol (mL)	14.4	9.35	<0.001
TPO-Ab (IU/mL)	442.65 ± 304.60	4.77 ± 5.75	<0.001
Tg-Ab (IU/mL)	701.68 ± 842.25	16.15 ± 19.14	<0.001
Se (μmol/L)	0.87 ± 0.29	1.11 ± 0.37	<0.001
GSH (μmol/L)	2.09 ± 2.06	6.35 ± 3.91	<0.001
GPX3 (IU/L)	326.29 ± 63.45	272.62 ± 44.33	<0.001
UIC (nmol/L)	985	780	NS
UIC/Cr (µmol/µmol)	11.36	6.27	<0.001

Data are presented as mean ± SD with the exception for TVol, UIC and UIC/Cr which are presented as medians. NS = not significant (*p* > 0.05).

## References

[1] Saranac L., Zivanovic S., Bjelakovic B., Stamenkovic H., Novak M., Kamenov B. (2011). Why is the thyroid so prone to autoimmune disease?. Horm. Res. Paediatr..

[2] Caturegli P., Kimura H., Rocchi R., Rose N.R. (2007). Autoimmune thyroid diseases. Curr. Opin. Rheumatol..

[3] Mazokopakis E.E., Chatzipavlidou V. (2007). Hashimoto’s thyroiditis and the role of selenium. Current concepts. Hell. J. Nucl. Med..

[4] Antonelli A., Ferrari S.M., Corrado A., Di Domenicantonio A., Fallahi P. (2015). Autoimmune thyroid disorders. Autoimmun. Rev..

[5] Duntas L.H. (2011). Environmental factors and thyroid autoimmunity. Ann. d’endocrinologie.

[6] Rasic-Milutinovic Z., Jovanovic D., Bogdanovic G., Trifunovic J., Mutic J. (2017). Potential influence of selenium, copper, zinc and cadmium on L-thyroxine substitution in patients with Hashimoto thyroiditis and hypothyroidism. Exp. Clin. Endocrinol. Diabetes..

[7] Duntas L.H. (2008). Environmental factors and autoimmune thyroiditis. Nat. Clin. Pract. Endocrinol. Metab..

[8] Zimmermann M.B., Köhrle J. (2002). The impact of iron and selenium deficiencies on iodine and thyroid metabolism: Biochemistry and relevance to public health. Thyroid.

[9] Mustacich D., Powis G. (2000). Thioredoxin reductase. Biochem. J..

[10] Duntas L.H. (2006). The role of selenium in thyroid autoimmunity and cancer. Thyroid..

[11] Chun O.K., Floegel A., Chung S.J., Chung C.E., Song W.O., Koo S.I. (2010). Estimation of antioxidant intakes from diet and supplements in US adults. J. Nutr..

[12] Shils M.E., Shike M. (2006). Modern Nutrition in Health and Disease.

[13] Sunde R.A., Bowman B.A., Russell R.M. (2006). Selenium. Present Knowledge in Nutrition.

[14] Socha K., Dziemianowicz M., Omeljaniuk W.J., Soroczyńska J., Borawska M.H. (2012). Nawyki żywieniowe a stężenie selenu w surowicy u pacjentów z chorobą Hashimoto. Probl. Hig. Epidemiol..

[15] Institute of Medicine (2005). Food and Nutrition Board, Dietary Reference Intakes: Energy, Carbohydrate, Fiber, Fat, Fatty Acids, Cholesterol, Protein and Amino Acids.

[16] Contempre B., Je D., Bebe N., Ch T., At D., Vanderpas J. (1991). Effect of selenium supplementation in hypothyroid subjects of an iodine and selenium deficient area: The possible danger of indiscriminate supplementation of iodine-deficient subjects with selenium. J. Clin. Endocrinol. Metab..

[17] Wu Q., Rayman M.P., Lv H., Schomburg L., Cui B., Gao C., Chen P., Guihua Z., Zhenan Z., Peng X. (2015). Low population selenium status is associated with increased prevalence of thyroid disease. J. Clin. Endocrinol. Metab..

[18] Huang Z., Rose A.H., Hoffmann P.R. (2012). The role of selenium in inflammation and immunity: From molecular mechanisms to therapeutic opportunities. Antioxid. Redox Signal..

[19] Metere A., Frezzotti F., Graves C.E., Vergine M., De Luca A., Pietraforte D., Giacomelli L. (2018). A possible role for selenoprotein glutathione peroxidase (GPx1) and thioredoxin reductases (TrxR1) in thyroid cancer: Our experience in thyroid surgery. Cancer Cell Int..

[20] Przybylik-Mazurek E., Zagrodzki P., Kuźniarz-Rymarz S., Hubalewska-Dydejczyk A. (2011). Thyroid disorders—Assessments of trace elements, clinical, and laboratory parameters. Biol. Trace Elem. Res..

[21] Nourbakhsh M., Ahmadpour F., Chahardoli B., Malekpour-Dehkordi Z., Nourbakhsh M., Hosseini-Fard S.R., Doustimotlagh A., Golestani A., Razzaghy-Azar M. (2016). Selenium and its relationship with selenoprotein P and glutathione peroxidase in children and adolescents with Hashimoto’s thyroiditis and hypothyroidism. J. Trace Elem. Med. Biol..

[22] Safaralizadeh R., Kardar G.A., Pourpak Z., Moin M., Zare A., Teimourian S. (2005). Serum concentration of selenium in healthy individuals living in Tehran. Nutr. J..

[23] Brunn J., Block U., Ruf G., Bos I., Kunze W.P., Scriba P.C. (1981). Volumetric analysis of thyroid lobes by real-time ultrasound (author’s transl.). Dtsch. Med. Wochenschr..

[24] Höfling D.B., Cerri G.G., Juliano A.G., Marui S., Chammas M.C. (2008). Value of thyroid echogenicity in the diagnosis of chronic autoimmune thyroiditis. Radiol. Bras..

[25] Gunter E.W., Lewis B.L., Koncikowski S.M. (1996). Laboratory Methods Used for the Third National Health and Nutrition Examination Survey (NHANES III).

[26] Morisi G., Patriarca M., Menotti A. (1988). Improved determination of selenium in serum by Zeeman atomic absorption spectrometry. Clin. Chem..

[27] Rostami R., Beiranvend A., Nourooz-Zadeh J. (2012). Nutritional iodine status in gestation and its relation to geographic features in Urmia County of northwest Iran. Food Nutr. Bull..

[28] Dunn J.T., Crutchfield H.E., Gutekunst R., Dunn A.D. (1993). Two simple methods for measuring iodine in urine. Thyroid.

[29] Rostami R., Aghasi M.R., Mohammadi A., Nourooz-Zadeh J. (2013). Enhanced oxidative stress in Hashimoto’s thyroiditis: Inter-relationships to biomarkers of thyroid function. Clin. Biochem..

[30] Naing L., Winn T., Rusli B.N. (2006). Practical issues in calculating the sample size for prevalence studies. Arch. Orofac. Sci..

[31] Dröge W., Breitkreutz R. (2000). Glutathione and immune function. Proc. Nutr. Soc..

[32] Moncayo R., Kroiss A., Oberwinkler M., Karakolcu F., Starzinger M., Kapelari K., Talasz H., Moncayo H. (2008). The role of selenium, vitamin C, and zinc in benign thyroid diseases and of selenium in malignant thyroid diseases: Low selenium levels are found in subacute and silent thyroiditis and in papillary and follicular carcinoma. BMC Endocr. Disord..

[33] Saiki M., Jaluul O., Sumita N.M., Vasconcellos M.B.A., Filho W.J. (2007). Trace element contents in serum of healthy elderly population of metropolitan Sao Paulo area in Brazil. J. Trace Elem. Med. Biol..

[34] Rayman M.P., Thompson A.J., Bekaert B., Catterick J., Galassini R., Hall E., Warren-Perry M., Beckett G.J. (2008). Randomized controlled trial of the effect of selenium supplementation on thyroid function in the elderly in the United Kingdom. Am. J. Clin. Nutr..

[35] Erdal M., Sahin M., Hasimi A., Uckaya G., Kutlu M., Saglam K. (2008). Trace element levels in hashimoto thyroiditis patients with subclinical hypothyroidism. Biol. Trace Elem. Res..

[36] McDonald C., Colebourne K., Faddy H.M., Flower R., Fraser J.F. (2013). Plasma selenium status in a group of Australian blood donors and fresh blood components. J. Trace Elem. Med. Biol..

[37] Wimmer I., Hartmann T., Brustbauer R., Minear G., Dam K. (2014). Selenium levels in patients with autoimmune thyroiditis and controls in lower Austria. Horm. Metab. Res..

[38] Maehira F., Luyo G.A., Miyagi I., Oshiro M., Yamane N., Kuba M., Nakazato Y. (2002). Alterations of serum selenium concentrations in the acute phase of pathological conditions. Clin. Chim. Acta.

[39] Federige M.A.F., Romaldini J.H., Miklos A.B.P.P., Koike M.K., Takei K., Portes E.D.S. (2017). Serum selenium and selenoprotein-P levels in autoimmune thyroid diseases patients in a select center: A transversal study. Arch. Endocrinol. Metab..

[40] Liu Y., Huang H., Zeng J., Sun C. (2013). Thyroid volume, goiter prevalence, and selenium levels in an iodine-sufficient area: A cross-sectional study. BMC Public Health.

[41] Feldt-Rasmussen U., Hegedüs L., Perrild H., Rasmussen N., Hansen J.M. (1989). Relationship between serum thyroglobulin, thyroid volume and serum TSH in healthy non-goitrous subjects and the relationship to seasonal variations in iodine intake. Thyroidology.

[42] Medina D.L., Santisteban P. (2000). Thyrotropin-dependent proliferation of in vitro rat thyroid cell systems. Eur. J. Endocrinol..

[43] Ozata M., Salk M., Aydin A., Sayin S., Oktenli C., Beyham Z., Isimer A., Ozdemir I.C. (1999). Iodine and zinc, but not selenium and copper, deficiency exists in a male Turkish population with endemic goiter. Biol. Trace Elem. Res..

[44] Mazokopakis E.E., Papadakis J.A., Papadomanolaki M.G., Batistakis A.G., Giannakopoulos T.G., Protopapadakis E.E., Ganotakis E.S. (2007). Effects of 12 months treatment with L-selenomethionine on serum anti-TPO Levels in Patients with Hashimoto’s thyroiditis. Thyroid.

[45] Karanikas G., Schuetz M., Kontur S., Duan H., Kommata S., Schoen R., Antoni A., Kletter K., Dudczak R., Willheim M. (2008). No immunological benefit of selenium in consecutive patients with autoimmune thyroiditis. Thyroid.

[46] Deponte M. (2013). Glutathione catalysis and the reaction mechanisms of glutathione-dependent enzymes. Biochim. Biophys. Acta (BBA)-Gen. Subj..

[47] Dominko K., Đikić D. (2018). Glutationilacija–regulacijska uloga glutationa u fiziološkim procesima. Arh. Hig. Rada Toksikol..

[48] Calder P.C. (2009). Polyunsaturated fatty acids and inflammatory processes: New twists in an old tale. Biochimie.

[49] Wu D., Meydani S.N., Sastre J., Hayek M., Meydani M. (1994). In vitro glutathione supplementation enhances interleukin-2 production and mitogenic response of peripheral blood mononuclear cells from young and old subjects. J. Nutr..

[50] Perricone C., De Carolis C., Perricone R. (2009). Glutathione: A key player in autoimmunity. Autoimmun. Rev..

[51] Hughes M.M., Mcgettrick A.F., O’Neill L.A.J. (2017). Glutathione and glutathione transferase omega 1 as key posttranslational regulators in macrophages. Microbiol. Spectr..

[52] Peterson J.D., Herzenberg L.A., Vasquez K., Waltenbaugh C. (1998). Glutathione levels in antigen-presenting cells modulate Th1 versus Th2 response patterns. Proc. Natl. Acad. Sci. USA.

[53] Hamilos D.L., Zelarney P., Mascali J.J. (1989). Lymphocyte proliferation in glutathione-depleted lymphocytes: Direct relationship between glutathione availability and the proliferative response. Immunopharmacology.

[54] Morris D., Khurasany M., Nguyen T., Kim J., Guilford F., Mehta R., Gray D., Saviola B., Venketaraman V. (2013). Glutathione and infection. Biochim. Biophys. Acta (BBA)-Gen. Subj..

[55] Richie J.P., Nichenametla S., Neidig W., Calcagnotto A., Haley J.S., Schell T.D., Muscat J.E. (2015). Randomized controlled trial of oral glutathione supplementation on body stores of glutathione. Eur. J. Nutr..

[56] Duntas L.H. (2010). Selenium and the thyroid: A close-knit connection. J. Clin. Endocrinol. Metab..

[57] Gamaley I.A., Klyubin I.V. (1999). Roles of reactive oxygen species: Signaling and regulation of cellular functions. Int. Rev. Cytol..

[58] Giuliani C., Bucci I., Napolitano G. (2018). The role of the transcription factor nuclear factor-kappa B in thyroid autoimmunity and cancer. Front. Endocrinol. (Lausanne).

[59] Carayanniotis G. (2011). Molecular parameters linking thyroglobulin iodination with autoimmune thyroiditis. Hormones (Athens).

[60] Sundick R.S., Bagchi N., Brown T.R. (1992). The role of iodine in thyroid autoimmunity: From chickens to humans: A review. Autoimmunity.

[61] Tsatsoulis A. (2018). The role of iodine vs selenium on the rising trend of autoimmune thyroiditis in iodine sufficient countries. Endocrinol. Int. J..

[62] Pirola I., Gandossi E., Agosti B., Delbarba A., Cappelli C. (2016). Selenium supplementation could restore euthyroidism in subclinical hypothyroid patients with autoimmune thyroiditis. Endokrynol. Pol..

[63] Wang W., Mao J., Zhao J., Lu J., Yan L., Du J., Lu Z., Wang H., Xu M., Bai X. (2018). Decreased thyroid peroxidase antibody titer in response to selenium supplementation in autoimmune thyroiditis and the influence of a selenoprotein P gene polymorphism: A prospective, multicenter study in China. Thyroid.

[64] Esposito D., Rotondi M., Accardo G., Vallone G., Conzo G., Docimo G., Selvaggi F., Cappelli C., Chiovato L., Giugliano D. (2017). Influence of short-term selenium supplementation on the natural course of Hashimoto’s thyroiditis: Clinical results of a blinded placebo-controlled randomized prospective trial. J. Endocrinol. Investig..

